# Meat Harmful in Summer

**Published:** 1893-06

**Authors:** 


### MEAT HARMFUL IN SUMMER.

Considering the excessive meat eating habit of our people diarrhea will doubtless be as usual prevalent during the present summer. Meat spoils very quickly in this climate in hot weather, and poisons are then developed in it which cause vomiting and purging. Some people are more susceptible to these poisons than others. Probably the most susceptible are the young and those adults who suffer from digestive disturbances in which the liver is involved and unable to do its work properly —in other words, those who are termed bilious. One office of the liver is to protect the system from poisoning. It destroys certain noxious agents, and particularly those of a putrid character. Of course there is a limit to this power in a healthy liver, and it must be materially lessened in one deranged. Those who cannot afford to keep their ice chests well filled should buy fresh meats only in small quantities, to be eaten at once. A more economical and safer plan is to forego meats entirely during the very hot weather, and depend upon fruits and vegetables. The vegetarian invariably suffers less discomfort and enjoys infinitely better health during the heated term than does the meat eater.

